# Tris(2,2′-bi-1*H*-imidazole-κ^2^ *N* ^3^,*N* ^3′^)cobalt(II) hydrogen phosphate

**DOI:** 10.1107/S1600536811037299

**Published:** 2011-09-17

**Authors:** Zhiqiang Liang, Fuxiang Wang, Qihui Wu, Xia Zhi, Qinhe Pan

**Affiliations:** a State Key Laboratory of Inorganic Synthesis and Preparative Chemistry, Jilin University, Changchun 130012, Jilin Province, People’s Republic of China; b Department of Materials and Chemical Engineering, Ministry of Education Key Laboratory of Application Technology of Hainan Superior Resources Chemical Materials, Hainan University, Haikou 570228, Hainan Province, People’s Republic of China

## Abstract

The title compound, [Co(C_6_H_6_N_4_)_3_]HPO_4_, was synthesized under hydro­thermal conditions. In the cation, the Co^II^ atom is octa­hedrally coordinated by six N atoms from three 2,2′-bi-1*H*-imidazole ligands [Co—N bond lengths are in the range 2.084 (5)–2.133 (6) Å]. Inter­molecular N—H⋯O hydrogen bonds form an extensive hydrogen-bonding network, which links cations and anions into a three-dimensional crystal structure.

## Related literature

For related compounds, see Pan *et al.* (2005 ▶, 2008 ▶, 2010*a* ▶,*b* ▶, 2011 ▶); Rothammel *et al.* (1998 ▶); Stalder & Wilkinson (1997 ▶); Tong & Pan (2011 ▶); Wang *et al.* (2003*a* ▶,*b* ▶).
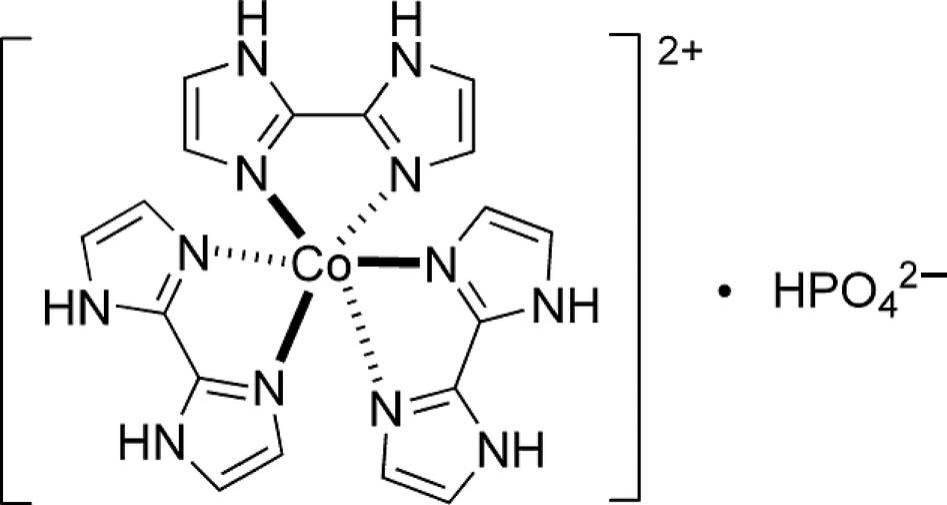


## Experimental

### 

#### Crystal data

[Co(C_6_H_6_N_4_)_3_]HPO_4_
*M* *_r_* = 557.35
Monoclinic, 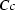

*a* = 12.700 (3) Å
*b* = 21.447 (4) Å
*c* = 9.1140 (18) Å
β = 95.84 (3)°
*V* = 2469.6 (8) Å^3^
*Z* = 4
Mo *K*α radiation
μ = 0.81 mm^−1^
*T* = 293 K
0.20 × 0.17 × 0.15 mm

#### Data collection

Rigaku R-AXIS RAPID-S diffractometer
Absorption correction: multi-scan (*CrystalClear*; Rigaku/MSC, 2002 ▶) *T*_min_ = 0.850, *T*_max_ = 0.886
12597 measured reflections
5593 independent reflections
3373 reflections with *I* > 2σ(*I*)
*R*_int_ = 0.098

#### Refinement

*R*[*F* ^2^ > 2σ(*F* ^2^)] = 0.077
*wR*(*F* ^2^) = 0.152
*S* = 1.06
5593 reflections
325 parameters
2 restraints
H-atom parameters constrained
Δρ_max_ = 0.40 e Å^−3^
Δρ_min_ = −0.32 e Å^−3^
Absolute structure: Flack (1983 ▶), 2755 Friedel pairs
Flack parameter: −0.02 (2)

### 

Data collection: *RAPID-AUTO* (Rigaku, 1998 ▶); cell refinement: *RAPID-AUTO*; data reduction: *CrystalStructure* (Rigaku/MSC, 2002 ▶); program(s) used to solve structure: *SHELXS97* (Sheldrick, 2008 ▶); program(s) used to refine structure: *SHELXL97* (Sheldrick, 2008 ▶); molecular graphics: *SHELXTL* (Sheldrick, 2008 ▶); software used to prepare material for publication: *SHELXTL*.

## Supplementary Material

Crystal structure: contains datablock(s) I, global. DOI: 10.1107/S1600536811037299/aa2021sup1.cif

Structure factors: contains datablock(s) I. DOI: 10.1107/S1600536811037299/aa2021Isup2.hkl

Additional supplementary materials:  crystallographic information; 3D view; checkCIF report

## Figures and Tables

**Table 1:** Hydrogen-bond geometry (Å, °)

*D*—H⋯*A*	*D*—H	H⋯*A*	*D*⋯*A*	*D*—H⋯*A*
N2—H2⋯O4	0.86	1.82	2.678 (7)	172.1
N4—H4⋯O2	0.86	1.87	2.717 (7)	168.3
N6—H6*A*⋯O3^i^	0.86	1.96	2.725 (8)	148.3
N8—H8⋯O3^i^	0.86	1.89	2.669 (8)	149.3
N10—H10⋯O2^ii^	0.86	2.23	2.887 (7)	133.7
N10—H10⋯O4^iii^	0.86	2.39	3.034 (9)	132.5
N12—H12*A*⋯O4^iii^	0.86	1.93	2.685 (8)	146.0

Symmetry codes: (i) 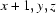; (ii) 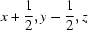; (iii) 
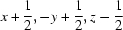
.

**Fig. 1.**
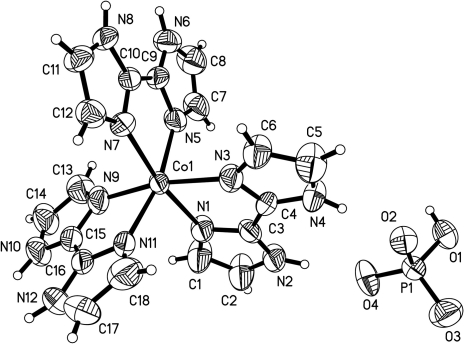
A view of the structure of complex. Ellipsoids are drawn at the 30% probability level.

## supplementary crystallographic information

### Comment

Recently, more attention has been paid to chiral metal complexes, which could be employed as an interesting template for the synthesis of novel materials, because they are versatile and can be made with a wide of shapes, charges and particularly chirality. Up to now, series of open-frameworks, such as metal phosphates (for example: Stalder & Wilkinson (1997); Wang *et al.* (2003*a*,*b*)) and germanates (for example: Pan *et al.* (2005, 2008)), have been synthesized with [*M*(*dien*)_2_]^n+^ or [*M*(*en*)_3_]^n+^ (*M* = Co, Ni; n = 2, 3; *dien* = diethylenediamine, *en* = ethylenediamine) cations. Recently the chiral metal complexes have been introduced into coordination polymers, see Pan *et al.* (2010*a*, 2010*b*, 2011), Tong *et al.* (2011). In this paper, we present an other metal complex [Co(*biim*)_3_]HPO_4_ (*biim* is 2,2'-biimidazole).

As shown in Fig. 1, the crystal structure of title compound consists of a discrete [Co(*biim*)_3_]^2+^ cations and HPO_4_^2-^ anions. In [Co(*biim*)_3_]^2+^, the Co^II^ center is six coorinated and linked by six N atoms from three different *biim* ligands, resulting in a slightly distorted octahedral geometry. The Co^II^—N bond distances are in the range of 2.084 (5)–2.133 (6) Å. The P atom displays a slightly distorted tetrahedal geometry and is bonded to three O atoms and one OH group with the P—O distances of 1.484 (6)–1.564 (5) Å. N—H···O hydrogen bonds connect cations and anions into a three-dimensional network (see Table 1).

### Experimental

In a typical synthesis, a mixture of Co(OAc)_2_.2H_2_O (0.25 g), biimidazole (0.067 g), H_3_PO_4_ (0.12 ml) and H_2_O (10 ml) were added to a 25 ml Teflon-lined reactor and kept under autogenous pressure at 120 °C for 3 days.

### Refinement

All H atoms were positioned geometrically (C—H = 0.93 Å, N—H = 0.86 Å and O—H = 0.82 Å) and allowed to ride on their parent atoms,with *U*_iso_(H) = 1.2*U*_eq_(parent atom).

### Crystal data

**Table:**

[Co(C_6_H_6_N_4_)_3_]HPO_4_	*F*(000) = 1140
*M**_r_* = 557.35	*D*_x_ = 1.499 Mg m^−^^3^
Monoclinic, *C**c*	Mo *K*α radiation, λ = 0.71073 Å
Hall symbol: C -2yc	Cell parameters from 12761 reflections
*a* = 12.700 (3) Å	θ = 3.1–27.5°
*b* = 21.447 (4) Å	µ = 0.81 mm^−^^1^
*c* = 9.1140 (18) Å	*T* = 293 K
β = 95.84 (3)°	Block, blue
*V* = 2469.6 (8) Å^3^	0.2 × 0.17 × 0.15 mm
*Z* = 4	

### Data collection

**Table:**

Rigaku R-AXIS RAPID-S diffractometer	5593 independent reflections
Radiation source: fine-focus sealed tube	3373 reflections with *I* > 2σ(*I*)
graphite	*R*_int_ = 0.098
ω scans	θ_max_ = 27.5°, θ_min_ = 3.1°
Absorption correction: multi-scan (*CrystalClear*; Rigaku/MSC, 2002)	*h* = −16→16
*T*_min_ = 0.850, *T*_max_ = 0.886	*k* = −27→27
12597 measured reflections	*l* = −11→11

### Refinement

**Table:**

Refinement on *F*^2^	Secondary atom site location: difference Fourier map
Least-squares matrix: full	Hydrogen site location: inferred from neighbouring sites
*R*[*F*^2^ > 2σ(*F*^2^)] = 0.077	H-atom parameters constrained
*wR*(*F*^2^) = 0.152	*w* = 1/[σ^2^(*F*_o_^2^) + (0.0453*P*)^2^] where *P* = (*F*_o_^2^ + 2*F*_c_^2^)/3
*S* = 1.06	(Δ/σ)_max_ = 0.042
5593 reflections	Δρ_max_ = 0.40 e Å^−^^3^
325 parameters	Δρ_min_ = −0.32 e Å^−^^3^
2 restraints	Absolute structure: Flack (1983), 2755 Friedel pairs
Primary atom site location: structure-invariant direct methods	Flack parameter: −0.02 (2)

### Special details

**Table:**

Geometry. All e.s.d.'s (except the e.s.d. in the dihedral angle between two l.s. planes) are estimated using the full covariance matrix. The cell e.s.d.'s are taken into account individually in the estimation of e.s.d.'s in distances, angles and torsion angles; correlations between e.s.d.'s in cell parameters are only used when they are defined by crystal symmetry. An approximate (isotropic) treatment of cell e.s.d.'s is used for estimating e.s.d.'s involving l.s. planes.
Refinement. Refinement of *F*^2^ against ALL reflections. The weighted *R*-factor *wR* and goodness of fit *S* are based on *F*^2^, conventional *R*-factors *R* are based on *F*, with *F* set to zero for negative *F*^2^. The threshold expression of *F*^2^ > σ(*F*^2^) is used only for calculating *R*-factors(gt) *etc*. and is not relevant to the choice of reflections for refinement. *R*-factors based on *F*^2^ are statistically about twice as large as those based on *F*, and *R*- factors based on ALL data will be even larger.

### Fractional atomic coordinates and isotropic or equivalent isotropic displacement parameters (Å^2^)

**Table:**

	*x*	*y*	*z*	*U*_iso_*/*U*_eq_	
Co1	0.61255 (7)	0.33459 (3)	0.15859 (9)	0.0466 (3)	
P1	0.12445 (13)	0.48617 (7)	0.34051 (17)	0.0397 (4)	
O1	0.1291 (4)	0.5348 (2)	0.4686 (5)	0.0560 (13)	
H1	0.1900	0.5375	0.5077	0.067*	
O2	0.1932 (4)	0.5113 (2)	0.2233 (4)	0.0512 (12)	
O3	0.0119 (4)	0.4852 (3)	0.2777 (6)	0.0828 (17)	
O4	0.1658 (4)	0.4249 (2)	0.4011 (5)	0.0699 (15)	
N1	0.4828 (5)	0.3189 (3)	0.2765 (7)	0.0619 (18)	
N2	0.3328 (5)	0.3553 (3)	0.3473 (7)	0.0648 (18)	
H2	0.2794	0.3792	0.3565	0.078*	
N3	0.5206 (5)	0.4153 (2)	0.0992 (6)	0.0513 (14)	
N4	0.3842 (4)	0.4723 (3)	0.1484 (6)	0.0566 (16)	
H4	0.3274	0.4838	0.1847	0.068*	
N5	0.7019 (4)	0.3812 (2)	0.3368 (6)	0.0513 (15)	
N6	0.8457 (5)	0.4352 (3)	0.4042 (7)	0.0584 (16)	
H6A	0.9047	0.4544	0.3993	0.070*	
N7	0.7434 (4)	0.3656 (3)	0.0530 (6)	0.0477 (14)	
N8	0.8950 (4)	0.4176 (2)	0.0763 (7)	0.0482 (14)	
H8	0.9473	0.4395	0.1150	0.058*	
N9	0.6751 (5)	0.2470 (3)	0.2311 (8)	0.0603 (17)	
N10	0.6751 (6)	0.1449 (3)	0.1916 (9)	0.074 (2)	
H10	0.6636	0.1093	0.1498	0.089*	
N11	0.5563 (4)	0.2775 (3)	−0.0218 (8)	0.0625 (18)	
N12	0.5553 (5)	0.1820 (3)	−0.1166 (9)	0.077 (2)	
H12A	0.5652	0.1426	−0.1244	0.092*	
C1	0.4432 (7)	0.2771 (4)	0.3717 (12)	0.095 (3)	
H1A	0.4748	0.2395	0.4015	0.114*	
C2	0.3535 (7)	0.2987 (4)	0.4145 (12)	0.097 (3)	
H2A	0.3118	0.2790	0.4787	0.116*	
C3	0.4129 (5)	0.3662 (3)	0.2641 (8)	0.0511 (18)	
C4	0.4347 (5)	0.4180 (3)	0.1736 (8)	0.0449 (17)	
C5	0.4392 (7)	0.5059 (4)	0.0548 (10)	0.078 (3)	
H5	0.4226	0.5454	0.0177	0.094*	
C6	0.5240 (6)	0.4697 (3)	0.0261 (9)	0.063 (2)	
H6	0.5756	0.4811	−0.0342	0.076*	
C7	0.7044 (7)	0.3963 (3)	0.4839 (9)	0.064 (2)	
H7	0.6529	0.3853	0.5447	0.077*	
C8	0.7923 (8)	0.4291 (4)	0.5266 (9)	0.071 (2)	
H8A	0.8128	0.4446	0.6205	0.086*	
C9	0.7899 (5)	0.4063 (3)	0.2959 (8)	0.0446 (16)	
C10	0.8109 (5)	0.3975 (3)	0.1443 (8)	0.0415 (16)	
C11	0.8805 (6)	0.3960 (3)	−0.0667 (8)	0.0520 (17)	
H11	0.9254	0.4018	−0.1400	0.062*	
C12	0.7866 (6)	0.3645 (3)	−0.0789 (8)	0.0562 (19)	
H12	0.7563	0.3452	−0.1643	0.067*	
C13	0.7277 (7)	0.2186 (4)	0.3508 (9)	0.070 (2)	
H13	0.7583	0.2388	0.4348	0.083*	
C14	0.7284 (8)	0.1565 (4)	0.3283 (12)	0.078 (3)	
H14	0.7593	0.1268	0.3933	0.094*	
C15	0.6450 (5)	0.2012 (3)	0.1372 (9)	0.057 (2)	
C16	0.5860 (6)	0.2179 (3)	0.0014 (10)	0.061 (2)	
C17	0.5049 (8)	0.2208 (5)	−0.2230 (12)	0.094 (3)	
H17	0.4756	0.2088	−0.3164	0.113*	
C18	0.5058 (7)	0.2797 (4)	−0.1671 (12)	0.086 (3)	
H18	0.4781	0.3151	−0.2158	0.103*	

### Atomic displacement parameters (Å^2^)

**Table:**

	*U*^11^	*U*^22^	*U*^33^	*U*^12^	*U*^13^	*U*^23^
Co1	0.0401 (4)	0.0300 (4)	0.0746 (6)	−0.0043 (5)	0.0292 (4)	−0.0064 (5)
P1	0.0473 (10)	0.0415 (9)	0.0323 (8)	−0.0023 (8)	0.0134 (8)	−0.0013 (8)
O1	0.075 (4)	0.048 (3)	0.046 (3)	0.005 (3)	0.012 (3)	−0.004 (2)
O2	0.057 (3)	0.051 (3)	0.048 (3)	−0.002 (2)	0.014 (2)	0.009 (2)
O3	0.058 (3)	0.100 (4)	0.091 (4)	−0.007 (3)	0.011 (3)	−0.023 (3)
O4	0.104 (4)	0.042 (3)	0.068 (3)	0.020 (3)	0.028 (3)	0.011 (2)
N1	0.053 (4)	0.043 (3)	0.098 (5)	−0.005 (3)	0.047 (4)	0.000 (3)
N2	0.055 (4)	0.047 (3)	0.100 (5)	−0.002 (3)	0.043 (4)	−0.002 (4)
N3	0.049 (3)	0.045 (3)	0.063 (4)	0.004 (3)	0.020 (3)	0.001 (3)
N4	0.047 (4)	0.060 (4)	0.064 (4)	0.018 (3)	0.013 (3)	0.009 (3)
N5	0.055 (4)	0.038 (3)	0.065 (4)	−0.004 (3)	0.026 (3)	−0.007 (3)
N6	0.059 (4)	0.054 (4)	0.064 (4)	−0.014 (3)	0.016 (4)	−0.006 (3)
N7	0.040 (3)	0.048 (3)	0.057 (4)	−0.006 (3)	0.016 (3)	−0.004 (3)
N8	0.036 (3)	0.044 (3)	0.066 (4)	−0.005 (2)	0.016 (3)	0.005 (3)
N9	0.057 (4)	0.046 (4)	0.084 (5)	0.002 (3)	0.038 (4)	−0.004 (4)
N10	0.079 (5)	0.034 (3)	0.115 (6)	−0.005 (3)	0.036 (5)	−0.005 (4)
N11	0.040 (3)	0.040 (4)	0.112 (6)	−0.001 (3)	0.026 (4)	−0.015 (4)
N12	0.063 (4)	0.038 (3)	0.129 (6)	−0.006 (3)	0.012 (4)	−0.022 (4)
C1	0.076 (6)	0.051 (5)	0.171 (9)	0.006 (4)	0.075 (7)	0.029 (6)
C2	0.084 (6)	0.053 (5)	0.167 (10)	0.006 (5)	0.082 (7)	0.029 (6)
C3	0.053 (4)	0.035 (4)	0.070 (5)	−0.008 (3)	0.028 (4)	−0.011 (4)
C4	0.030 (4)	0.046 (4)	0.060 (5)	0.002 (3)	0.013 (3)	−0.007 (3)
C5	0.086 (6)	0.070 (6)	0.084 (6)	0.015 (5)	0.037 (6)	0.031 (5)
C6	0.060 (5)	0.058 (5)	0.077 (5)	0.006 (4)	0.033 (4)	0.022 (4)
C7	0.079 (6)	0.056 (5)	0.062 (5)	−0.003 (4)	0.031 (5)	−0.009 (4)
C8	0.089 (6)	0.069 (6)	0.060 (5)	−0.010 (5)	0.025 (5)	−0.010 (4)
C9	0.037 (4)	0.038 (4)	0.060 (5)	−0.001 (3)	0.014 (4)	0.006 (3)
C10	0.043 (4)	0.032 (3)	0.052 (4)	−0.002 (3)	0.019 (4)	0.002 (3)
C11	0.043 (4)	0.063 (5)	0.052 (4)	−0.009 (4)	0.016 (4)	0.000 (4)
C12	0.061 (5)	0.055 (5)	0.053 (5)	−0.007 (4)	0.009 (4)	−0.007 (4)
C13	0.080 (6)	0.058 (5)	0.074 (6)	−0.006 (4)	0.023 (5)	0.004 (4)
C14	0.090 (7)	0.042 (5)	0.107 (8)	0.007 (4)	0.029 (6)	0.005 (5)
C15	0.046 (4)	0.035 (4)	0.094 (6)	−0.003 (3)	0.027 (4)	−0.002 (4)
C16	0.041 (4)	0.035 (4)	0.111 (7)	−0.005 (3)	0.031 (5)	−0.009 (5)
C17	0.081 (7)	0.064 (6)	0.134 (9)	−0.013 (5)	−0.007 (6)	−0.029 (6)
C18	0.059 (6)	0.079 (7)	0.116 (8)	−0.001 (5)	−0.006 (6)	0.003 (6)

### Geometric parameters (Å, °)

**Table:**

Co1—N1	2.084 (5)	N9—C15	1.332 (9)
Co1—N7	2.110 (5)	N9—C13	1.364 (10)
Co1—N11	2.115 (7)	N10—C15	1.346 (9)
Co1—N9	2.120 (6)	N10—C14	1.379 (11)
Co1—N3	2.127 (6)	N10—H10	0.8600
Co1—N5	2.133 (6)	N11—C16	1.344 (9)
P1—O3	1.485 (6)	N11—C18	1.412 (11)
P1—O4	1.500 (4)	N12—C16	1.348 (10)
P1—O2	1.544 (4)	N12—C17	1.384 (11)
P1—O1	1.563 (5)	N12—H12A	0.8600
O1—H1	0.8200	C1—C2	1.325 (10)
N1—C3	1.344 (9)	C1—H1A	0.9300
N1—C1	1.378 (9)	C2—H2A	0.9300
N2—C3	1.350 (8)	C3—C4	1.429 (9)
N2—C2	1.373 (9)	C5—C6	1.373 (10)
N2—H2	0.8600	C5—H5	0.9300
N3—C4	1.343 (8)	C6—H6	0.9300
N3—C6	1.347 (8)	C7—C8	1.343 (11)
N4—C4	1.337 (8)	C7—H7	0.9300
N4—C5	1.363 (9)	C8—H8A	0.9300
N4—H4	0.8600	C9—C10	1.446 (9)
N5—C9	1.327 (8)	C11—C12	1.365 (9)
N5—C7	1.376 (8)	C11—H11	0.9300
N6—C9	1.311 (8)	C12—H12	0.9300
N6—C8	1.370 (9)	C13—C14	1.347 (10)
N6—H6A	0.8600	C13—H13	0.9300
N7—C10	1.323 (8)	C14—H14	0.9300
N7—C12	1.372 (8)	C15—C16	1.426 (11)
N8—C10	1.358 (8)	C17—C18	1.361 (11)
N8—C11	1.377 (9)	C17—H17	0.9300
N8—H8	0.8600	C18—H18	0.9300
			
N1—Co1—N7	170.6 (2)	C16—N12—H12A	126.5
N1—Co1—N11	94.9 (2)	C17—N12—H12A	126.5
N7—Co1—N11	92.7 (2)	C2—C1—N1	109.8 (7)
N1—Co1—N9	89.3 (2)	C2—C1—H1A	125.1
N7—Co1—N9	97.7 (2)	N1—C1—H1A	125.1
N11—Co1—N9	79.4 (3)	C1—C2—N2	108.0 (7)
N1—Co1—N3	79.6 (2)	C1—C2—H2A	126.0
N7—Co1—N3	93.7 (2)	N2—C2—H2A	126.0
N11—Co1—N3	98.0 (2)	N1—C3—N2	110.6 (6)
N9—Co1—N3	168.4 (2)	N1—C3—C4	118.0 (6)
N1—Co1—N5	94.0 (2)	N2—C3—C4	131.3 (7)
N7—Co1—N5	79.5 (2)	N4—C4—N3	110.6 (6)
N11—Co1—N5	167.1 (2)	N4—C4—C3	131.2 (6)
N9—Co1—N5	91.4 (2)	N3—C4—C3	118.2 (6)
N3—Co1—N5	92.8 (2)	N4—C5—C6	106.4 (6)
O3—P1—O4	114.6 (4)	N4—C5—H5	126.8
O3—P1—O2	109.2 (3)	C6—C5—H5	126.8
O4—P1—O2	111.0 (3)	N3—C6—C5	109.3 (6)
O3—P1—O1	105.1 (3)	N3—C6—H6	125.4
O4—P1—O1	109.0 (3)	C5—C6—H6	125.4
O2—P1—O1	107.6 (3)	C8—C7—N5	110.0 (7)
P1—O1—H1	109.5	C8—C7—H7	125.0
C3—N1—C1	105.3 (6)	N5—C7—H7	125.0
C3—N1—Co1	112.7 (5)	C7—C8—N6	106.3 (7)
C1—N1—Co1	142.0 (5)	C7—C8—H8A	126.9
C3—N2—C2	106.4 (6)	N6—C8—H8A	126.9
C3—N2—H2	126.8	N6—C9—N5	112.7 (6)
C2—N2—H2	126.8	N6—C9—C10	130.0 (6)
C4—N3—C6	106.3 (6)	N5—C9—C10	117.3 (6)
C4—N3—Co1	111.1 (4)	N7—C10—N8	111.5 (6)
C6—N3—Co1	141.9 (5)	N7—C10—C9	119.8 (6)
C4—N4—C5	107.4 (6)	N8—C10—C9	128.7 (7)
C4—N4—H4	126.3	C12—C11—N8	106.0 (6)
C5—N4—H4	126.3	C12—C11—H11	127.0
C9—N5—C7	104.0 (6)	N8—C11—H11	127.0
C9—N5—Co1	112.0 (5)	C11—C12—N7	110.2 (6)
C7—N5—Co1	144.1 (5)	C11—C12—H12	124.9
C9—N6—C8	107.0 (6)	N7—C12—H12	124.9
C9—N6—H6A	126.5	C14—C13—N9	109.3 (8)
C8—N6—H6A	126.5	C14—C13—H13	125.4
C10—N7—C12	105.5 (5)	N9—C13—H13	125.4
C10—N7—Co1	111.5 (4)	C13—C14—N10	107.8 (8)
C12—N7—Co1	143.0 (5)	C13—C14—H14	126.1
C10—N8—C11	106.8 (6)	N10—C14—H14	126.1
C10—N8—H8	126.6	N9—C15—N10	111.8 (8)
C11—N8—H8	126.6	N9—C15—C16	117.7 (7)
C15—N9—C13	105.7 (6)	N10—C15—C16	130.5 (7)
C15—N9—Co1	112.2 (6)	N11—C16—N12	111.3 (8)
C13—N9—Co1	141.6 (6)	N11—C16—C15	119.3 (7)
C15—N10—C14	105.5 (7)	N12—C16—C15	129.3 (7)
C15—N10—H10	127.3	C18—C17—N12	108.0 (9)
C14—N10—H10	127.3	C18—C17—H17	126.0
C16—N11—C18	105.8 (7)	N12—C17—H17	126.0
C16—N11—Co1	111.2 (6)	C17—C18—N11	107.9 (8)
C18—N11—Co1	142.7 (6)	C17—C18—H18	126.1
C16—N12—C17	107.0 (7)	N11—C18—H18	126.1

### Hydrogen-bond geometry (Å, °)

**Table:**

*D*—H···*A*	*D*—H	H···*A*	*D*···*A*	*D*—H···*A*
N2—H2···O4	0.86	1.82	2.678 (7)	172.1
N4—H4···O2	0.86	1.87	2.717 (7)	168.3
N6—H6A···O3^i^	0.86	1.96	2.725 (8)	148.3
N8—H8···O3^i^	0.86	1.89	2.669 (8)	149.3
N10—H10···O2^ii^	0.86	2.23	2.887 (7)	133.7
N10—H10···O4^iii^	0.86	2.39	3.034 (9)	132.5
N12—H12A···O4^iii^	0.86	1.93	2.685 (8)	146.0

Symmetry codes: (i) *x*+1, *y*, *z*; (ii) *x*+1/2, *y*−1/2, *z*; (iii) *x*+1/2, −*y*+1/2, *z*−1/2.
